# Promoting Translational and Clinical Science: The Critical Role of Medical Schools and Teaching Hospitals

**DOI:** 10.1371/journal.pmed.0030378

**Published:** 2006-09-05

**Authors:** Howard B Dickler, David Korn, Steven G Gabbe

## Abstract

Academic translational and clinical research in the US faces serious obstacles. Dickler and colleagues outline the Association of American Medical Colleges recommendations for overcoming those barriers.

Historic advances in biomedical research provide both unprecedented insights into the pathogenesis of human diseases and the challenge to translate these insights efficiently into improved health. Meeting this challenge requires the creation of a robust translational and clinical research enterprise [1], which, in turn, depends critically on the ability of academic medicine to produce and support enough physician–scientists to enable the enterprise.

Yet, academic translational and clinical research in the United States continues to face serious obstacles. These obstacles include the sharply diminishing ability of clinical departments to underwrite research from patient-care revenues; a declining share of industry-sponsored clinical trials; the prospect of flat or declining National Institutes of Health (NIH) budgets over the near term; the perceived disadvantage of clinical research applications in NIH peer review; overly burdensome regulatory requirements; undervaluation by academic medical culture; and the lure of greater financial rewards, and even professional satisfaction, in full-time clinical practice.

Responding to this challenge, the Association of American Medical Colleges convened its second Clinical Research Task Force (CRTF II) to advise the academic medical community how best to (1) attract, develop, and nurture increased numbers of independent translational and clinical investigators; (2) create infrastructure needed for these investigators to be successful; and (3) finance translational and clinical science.

This Policy Forum summarizes the task force’s recommendations and conclusions, which are animated by the vision of an academic medical culture that values and supports translational and clinical research; transmits the excitement of such research to medical students, residents, and postdoctoral fellows; and establishes viable and

Academic translational and clinical research continues to face serious obstacles.

appealing career paths for physician investigators who conduct such research. The full report, which is available both in monograph and on the Web [2], focuses specifically on the obstacles facing academic translational and clinical research in the US; a recent report addresses related issues in the United Kingdom [3].

## Developing and Nurturing Translational and Clinical Physician–Scientists

### Education

Every future physician should receive a thorough education in the basic principles of translational and clinical research, both in medical school and during residency training. The Liaison Committee on Medical Education (the accrediting body for all US allopathic medical schools) should add education in translational and clinical research to the requirements for medical school accreditation, and the Accreditation Council for Graduate Medical Education (the accrediting body for specialty training programs) should embed the understanding of translational and clinical research within its required core competencies. By choosing these recommendations to open its report, the task force underscores the importance of requiring all future physicians to be educated in the principles and methodologies of translational and clinical research. This would send a clear signal that the institution’s leadership and faculty consider such research part of the core mission of academic medicine, and understanding it, a foundational element of medical education. The requirement would, at minimum, better equip physicians to (1) read the medical literature and evaluate the significance and implications of published discoveries and new treatments in their own disciplines, which is essential for the realization of evidence-based medicine; (2) communicate knowledgeably with clinical researchers; (3) explain translational and clinical research in a way that is comprehensible to their patients and refer them, when appropriate, for screening for clinical trials; and (4) be powerful advocates for translational and clinical research with the public. These are all appropriate objectives for medical school graduates irrespective of their career choices.

Equally important, early exposure of medical students to translational and clinical research will enhance their awareness of it and stimulate some to consider making it their career. To put teeth into Recommendation 1, CRTF II urges the two US accrediting organizations that oversee the formal phases of medical education to incorporate meaningful exposure to translational and clinical research into their accreditation standards.

### Training

Training for translational and clinical investigators should comprise the completion of an advanced degree with a thesis project (or equivalent educational experience), appropriate mentoring, and a substantive postdoctoral training experience. Translational and clinical research has grown sufficiently complex that training should include a degree program (at minimum, Master’s level) with a core curriculum and mentored thesis project, as well as an additional two to three years of rigorous, mentored postdoctoral experience to prepare trainees for independence. Providing trainees with opportunities to develop a record of accomplishment will enhance their attractiveness as candidates for junior faculty appointments. A foundational element of this recommendation is the need for institutional support of mentors, who are essential for success.

### Support

Sufficient support should be given to new junior faculty who are translational and clinical investigators to maximize their probability of success. New junior faculty in translational and clinical science require start-up support comparable to that which is provided in basic science—i.e., sufficient to establish an independent research program. This support includes space for research, salary support to protect ample time for research (often 75%), and resources to access the necessary infrastructure—e.g., biostatistics, study coordinators, laboratory and imaging cores, and informatics. Because of the complex nature and regulatory requirements of translational and clinical research, junior faculty continue to need effective, individually focused mentoring, and this should be overseen by the institution. Institutional policy must also be flexible enough to address the special needs of faculty who wish to start families.

### Accelerated training

Training in translational and clinical research should be accelerated through comprehensive re-structuring to enable trainees to become independent clinicians and investigators at the earliest possible time. The average age to receive one’s first R01 grant (investigator-initiated research project grant) from the NIH has been steadily increasing, and is now 42 years old for a PhD and 43 years old for an MD. The CRTF II is especially troubled by the effect that this delay in achieving independent funding has on recruitment into translational and clinical research. A number of US institutions have incorporated elements of translational and clinical research training—including Master’s degree programs—into medical school, residencies, or fellowships without adding significantly to the total amount of time in training. However, the task force envisions more fundamental restructuring that would begin early in medical school and continue seamlessly through residency and fellowship training to prepare graduates to compete for independent research funding at an earlier age. This would permit graduates to begin contributing to science during their years of highest creativity, and have more productive years in their careers. The Association of American Medical Colleges intends to facilitate such a reengineering effort.

### Academic recognition

Institutions, journals, the NIH, and other research sponsors should take steps to facilitate appropriate academic recognition of translational and clinical scientists for their contributions to collaborative research. Career advancement of academic translational and clinical scientists requires that they be given appropriate credit for their roles in conceptualizing, implementing, and obtaining research funding for published research. This becomes especially important as biomedical research becomes more multidisciplinary and team oriented. All journals should require (as some already do) a description of each author’s contribution to submitted manuscripts, and this information should be published with the article or should be accessible online. The NIH should modify its Computer Retrieval of Information on Scientific Projects (the NIH’s comprehensive online database of all NIH-funded grants) to list, in a searchable fashion, the roles of all co-investigators in funded grants and contracts.

### K23 and K24 awards

The NIH should modify the K23 and K24 awards to enhance their value in supporting clinical and translational research training and mentoring. K23 grants are mentored patient-oriented career development awards that provide partial salary support for clinical physician research trainees. K24 grants are mid-career patient-oriented research awards that provide salary support for 50% effort by clinical physician investigators for mentoring and conducting clinical research. CRTF II believes that all translational and clinical research trainees supported by NIH K23 grants should be supported for their stipulated effort at salary rates up to the legislative cap (US$183,500 in 2006), and that the awards should provide substantial support for the trainees’ own projects. Acknowledging the NIH’s presently constrained budget, CRTF II believes that fewer awards modified as recommended would better serve the goal of preparing trainees for independent careers.

The NIH should modify the K24 grants to allow any investigators who are independently funded by the NIH for patient-oriented research to apply, by permitting flexibility in the acceptable level of effort for mentoring (10%–50%). This would permit many more patient-oriented investigators to become eligible and likely take advantage of this support.

## Administration and Infrastructure for Translational and Clinical Science

### Oversight, administration, and support

Institutions should provide central oversight, administration, and support for the essential infrastructure required by the translational and clinical research enterprise. Centralized institutional leadership can powerfully enable translational and clinical research by promoting a culture in which translational and clinical research is vibrant and visible, strengthening the identity and morale of translational and clinical scientists, enabling strategic planning and targeted investment of resources, and promoting cohesion among the various components and partnerships across medical school departments and other schools. Centralized oversight and support of core resources will enhance usage by providing fair, unimpeded access for any faculty member who needs their services, as well as operational advantages. Such advantages include efficiency, cost savings, continuity of funding, availability of backup personnel, uniform operating procedures and training that improve compliance, and uniform standards for the qualifications and experience of support personnel.

### Human research protection programs

Human research protection programs (institutional programs that provide program support including training, administration, and quality control for the human research protections functions including the institutional review boards [IRBs; research ethics committees]) should be made more effective and efficient by (1) transagency harmonization of federal regulations, (2) accreditation of human research protection programs, (3) simplification of institutional regulatory compliance processes, and (4) expanded use of central IRBs (research ethics committees) in multisite research. US regulations pertinent to translational and clinical research and compliance should be simplified and harmonized between the NIH and the Food and Drug Administration. Institutions should seek accreditation for their human research protection programs: this will help establish an institutional “culture of responsibility,” lead to adoption of operating procedures that meet a national standard and are consistent across the academic medical community, and provide uniform education for faculty, trainees, and staff.

To minimize regulatory burdens on faculty, institutions should strive to create process improvements, e.g., a common renewal date for all compliance approvals required for each research study. To expedite approval of protocols and mitigate the burdens on individual IRBs and faculty time, as well as faculty frustration, academic institutions should make greater use of commercial, governmentally established, or institutionally designated central IRBs, especially for multisite trials. Wide adoption of human research protection program accreditation could help to promote this change.

### National forum

A national forum should be established to (1) facilitate development of clinical information systems that integrate data from diverse clinical and research information platforms, and to (2) develop DNA and tissue banks that correlate genotypic and phenotypic data and ensure regulatory compliance. Enabling translational and clinical research to reach its full potential, while keeping costs manageable, will require (1) the development of costly new generations of clinical information systems that can link clinical research databases across different platforms, and (2) the establishment of DNA and human tissue banks that adhere to common, rigorous operating standards, that are compliant with regulatory requirements, and that can enable correlation of genotypic and phenotypic information. These objectives could be accomplished most rapidly with minimum duplication by establishing a national forum in which government, industry, and institutions can share knowledge, information, and resources. One potential goal for a national forum would be to enumerate the unique information needs of clinical research databases that distinguish them from existing electronic medical records.

### Collaborations

Academic medical institutions should establish collaborations with community health-care providers and practice-based research networks to broaden the diversity and size of the population base for translational and clinical research and to increase opportunities for health services, epidemiological, and outcomes research. The US is rapidly becoming a nation of “minorities”; in the relatively near future, no ethnic or racial group in the country will exceed 50% of the population. Medical research will fail to reach its full promise and potential if it is not conducted by an appropriately diverse workforce in representative populations. The recruitment of sufficient numbers of appropriate research participants will be impossible without developing genuine partnerships with communities and local health-care providers. More effective linkages among academic medical centers, their Veterans Administration Medical Center affiliates, community physicians, and practice-based networks will require institutions to invest in infrastructure, as well as in training for faculty, staff, and students in the principles and challenges of community-based research. Forming collaborative relationships will require institutions to demonstrate their ability to accommodate the interests of communities and their physicians, as well as those of faculty, such that all participants perceive benefit.

## Financing for Translational and Clinical Science

Medical schools and their affiliated teaching hospitals should explicitly recognize and vigorously promote translational and clinical research as a core mission, and grant it a high priority for institutional funding. Leaders of institutions that have had success in developing translational and clinical science highly value this field of science, and visibly foster a supportive institutional culture. The leadership of every medical school has discretionary funds to invest in its missions, and institutions that value translational and clinical research make it a high priority for funding. Support is provided for trainees, junior faculty, and core infrastructure to create viable and appealing career paths for translational and clinical investigators. Successful institutions do not need large endowments or resources—impressive translational and clinical research programs can be built by targeting investments in focused areas, leveraging available resources through sponsored programs, and maximizing collaborations.

## Conclusion

Academic medicine has played a central role in partnering with the NIH and Industry to make the 21st century the “Century of Biology.” Now, academic medicine must also vigorously assert its leadership in translating that biology into better health for all.

## Abbreviations

CRTF II: Clinical Research Task Force
IRB: institutional review board
NIH: National Institutes of Health
